# Synergistic Regulatory Effect of Inhibin and Anti-Müllerian Hormone on Fertility of Mice

**DOI:** 10.3389/fvets.2021.747619

**Published:** 2021-10-28

**Authors:** Xue Yu, Tong Qiao, Liping Hua, Shuanghang Liu, Xinzhe Zhao, Ce Lv, Xuhong Zhao, Jing Wang, Li Han, Liguo Yang, Aixin Liang

**Affiliations:** ^1^Key Laboratory of Agricultural Animal Genetics, Breeding and Reproduction of Ministry of Education, College of Animal Science and Technology, Huazhong Agricultural University, Wuhan, China; ^2^Shandong Provincial Key Laboratory of Biophysics, Institute of Biophysics, Dezhou University, Dezhou, China; ^3^College of Veterinary Medicine, Huazhong Agricultural University, Wuhan, China

**Keywords:** INHA, AMH, granulosa cells, litter size, mice

## Abstract

Inhibin (INH) and anti-Müllerian hormone (AMH) are essential in ovarian folliculogenesis and play an inhibitory role in mammalian fertility. However, the interactive effect of INH and AMH on the animal reproduction remains unknown. This study aimed to determine the possible interaction and synergy between INH and AMH in steroidogenesis by primary granulosa cells, and investigate their synergistic effect on fertility in mice. In *in vitro* granulosa cell culture system, we found that the treatment of either INHA or AMH had no significant effect on basal estradiol and progesterone production, whereas both significantly attenuated FSH-induced steroid hormone secretion. Importantly, combined treatment with INHA and AMH showed additive inhibitory effect on FSH-induced estradiol and progesterone production, accompanying a significant downregulation in the expression of FSH-stimulated *CYP19A1, HSD3B, CYP11A1, StAR* transcripts. The interrelationship of INH and AMH combinations was further investigated through active immune neutralization strategy. Female mice were immunized against INH and AMH eukaryotic expression plasmids, and the litter size was recorded after successfully mating. We observed that both INH and AMH plasmids were able to induce either anti-AMH or anti-INH antibodies in the immunized mice. In comparison with the control group, co-immunization with INH and AMH plasmids induced higher levels of estradiol, resulting in more litter size. Moreover, there was no significant difference on the offspring's weight between each group. Collectively, the results of the present study suggest that INH and AMH have synergistic effect in regulating steroidogenesis and the litter size in mice.

## Introduction

The fundamental functions of the ovary are to produce oocytes and steroid hormones, which are mainly under the control of two glycoprotein hormones follicle-stimulating hormone (FSH) and luteinizing hormone (LH). Within the microenvironment of the ovary, local growth factors seem to play a crucial role in regulation of granulosa cell proliferation and differentiation, oocyte maturation, as well as steroidogenic activity. Among these factors, inhibin (INH) and anti-Müllerian hormone (AMH) are the members of the transforming growth factor β (TGF-β) superfamily, which is known to be the prime regulators of ovarian function in both autocrine or paracrine manner.

INH is a heterodimer glycoprotein consisting of two subunits, an α-subunit and either of two related β-subunits (βA and βB), thus, forming INHA (α-βA) and INHB (α-βB) (1– 3). INHA expresses mainly in pre-ovulatory follicles, while INHB exists in the intermediately antral follicles (4). INH has long been regarded as a suppressor of FSH secretions through pituitary–gonadal negative feedback to regulate ovarian functions (5, 6). In transgenic mice model, it has been demonstrated that INH α-subunit leads to the disruption of the normal INH-to-activin ratio and reproductive deficiencies (7). In theca cells, INH has been reported to decrease the inhibitory effects of activin on the expression of *StAR* and *HSD3B* and androgen production (8). However, its role in steroidogenesis of granulosa cells remains equivocal. INH has been revealed to decrease FSH-induced estradiol production and aromatase activity by granulosa cells in rat (6, 9) and cattle (10). In contrast, INH A promotes the estradiol production in sheep granulosa cells, and the antiserum of INH can attenuate FSH-stimulated estradiol production (11).

On the other hand, anti-Müllerian hormone (AMH), another member of the TGF-β superfamily, is mainly expressed in the gonads and plays an important role in the sexual differentiation and gonadal function. In the ovary, the expression of AMH begins in the primary follicles, which is the highest in the preantral follicles and small follicles, and decreases with the increase in antral follicle diameter in mice (12, 13), rats (14), humans (15, 16), cattle (17), and buffaloes (18). AMH has been clearly demonstrated to be involved in inhibiting the primordial follicle recruitment (19) and the growth of preantral and antral follicles through regulating the sensitivity of FSH (20). Furthermore, AMH abolishes FSH-dependent aromatase and estradiol production through its specific type II receptor (AMHRII) by sharing BMP signaling pathway in granulosa cells (21–23). In addition, AMH has been recognized as a reliable marker for ovarian reserve (24, 25), superovulation response (17, 26), and outcome of *in vitro* embryos (27–29).

In the clinics, both AMH and INHA are used as markers to diagnose polycystic ovary syndrome (PCOS), and the sensitivity reached 96.2% when both AMH and INH are detected in combination (30), indicating that AMH and INHA may have complementary roles in abnormal ovarian function. However, the possible interactive action of AMH and INH in ovarian function has not yet been determined. In the current study, we aimed to explore the synergistic regulatory effect of AMH and INH on steroidogenesis in the presence/absence of FSH by primary granulosa cells and the fertility including litter size and steroid hormones in female mice.

## Materials and Methods

### Cell Culture

Three-week-old female mice were intraperitoneally injected with 10 IU of pregnant mare serum gonadotropin *(PMSG, Ningbo Sansheng Pharmaceutical Corporation, China)* to stimulate granulosa cell proliferation. The mice were then sacrificed 48 h later by cervical dislocation. Ovaries were carefully removed and placed in a sterile 35-mm cell culture dish (five to six ovaries per dish) containing 2 ml of DMEM/F12 medium *(HyClone, USA)*. The granulosa cells (GCs) were harvested by repeatedly puncturing the antral follicles with a sterile 25-gauge needle. Follicular fluid was filtered by a 40-μm nylon cell strainer, and then centrifuged at 700 × *g* for 5 min to obtain the GCs in the form of pellets. GC pellets were then washed twice in 6-ml DMEM/F12 followed by centrifugation at 700 × *g* for 5 min. After a final wash, the cells were seeded into 6-well plates at a density of 1 × 10^6^ cells/ml in DMEM/F12 medium supplemented with 10% fetal bovine serum *(FBS, Gibco, USA)*, 100 IU/ml of penicillin *(Gibco, USA)*, and 100 μg/ml of streptomycin *(Gibco, USA)*, and incubated in a humidified 5% CO_2_ at 37°C.

### cAMP Assay

Granulosa cells were treated either alone or in combination with increasing concentrations of INHA (*R* & *D Systems, USA*) and AMH (*R* & *D Systems, USA*) in the presence or absence of 100 ng/ml of FSH (*R* & *D Systems, USA*) for 30 min, and then the cell lysates were collected. IBMX *(Sigma-Aldrich, USA)* served as an inhibitor to avoid a drop in the levels of cAMP within the granulosa cells. cAMP accumulation was measured using a cAMP Parameter Assay kit (*R* & *D Systems, USA*) according to the protocol of the manufacturer.

### Detection of Estradiol and Progesterone in the Supernatant

For the detection of estradiol production, 100 nM androstenedione *(Sigma-Aldrich, USA)*, a substrate of CYP19, was supplemented to each GC culture medium. Granulosa cells were cultured with INHA or AMH alone or in combination with INHA and AMH in the presence or absence of FSH for 48 h. The levels of steroid hormones in the supernatant were measured according to estradiol *(Cusabio, CSB-E05109m, China)* and progesterone *(Cusabio, CSB-E05104m, China)* ELISA kit instructions. The assay sensitivity was 40 pg/ml for estradiol and 0.2 ng/ml for the progesterone.

### Reverse Transcription and Quantitative PCR

After treatment with INHA and AMH alone or in combination for 48 h, total RNA was extracted with E.Z.N.A Total RNA Kit I *(Omega, R6834-02, USA)*. cDNA was synthesized from 500 ng RNA of each samples by Quanti TectReverse Transcription Kit *(QIAGEN, Germany)*. The quantitative PCR was performed using Quantinova SYBR Green PCR Kit *(QIAGEN, Germany)* on CFX384 real-time PCR detection system *(Bio-Rad, USA)*. Specific primers were designed by Primer Premier 5.0 and shown in Table 1. A total of 10-μl reaction solution composed of 5 μl of SYBR Green Mix, 1 μl of primers, 3 μl of H_2_O, and 1 μl of diluted cDNA (1:10 for CYP11A1, HSD3B, StAR; 1:1 for CYP19A1). All samples were run under the following conditions: 95°C for 5 min, 39 cycles of amplifications (95°C for 20 s, 60°C for 20 s, 72°C for 20 s). Melting curve analysis was performed in the range of 65 to 95°C, 0.5°C per 5-s increments. The relative expression of genes was displayed as 2^−ΔΔCT^ normalized by β-actin.

**Table 1:** Primers for qRT-PCR in this study.

**Gene**	**Sequence (5′-3′)**	**Amplicon size (bp)**	**Accession no**.
CYP19A1	F:CCCGAGCCTTTGGAGAACAA	161	NM_001348171.1
	R:TGAGGGTCAACACATCCACG		
StAR	F:GTGAAGGCTAAGGGATAA	125	NM_011485.5
	R:TGGAGCTGGTAAGACAAC		
CYP11A1	F:ACATGGCCAAGATGGTACAGTTG R:ACGAAGCACCAGGTCATTCAC	119	NM_001346787.1
HSD3B	F:TGGACAAAGTATTCCGACCAGA R:GGCACACTTGCTTGAACACAG	250	NM_008293
β-actin	F: TAAAGACCTCTATGCCAACACAGT R:CACGATGGAGGGGCCGGACTCATC	241	NM_007393.5

### Plasmid Preparation and Immunization

The recombinant plasmids, pVAX-tPA-SINH-asd and pVAX-tPA-SAMH-asd were constructed and preserved by our laboratory. Briefly, pVAX-tPA-SINH-asd encodes SINH gene fusing with INH subunit α (1–32) and hepatitis B surface antigen S gene, while pVAX-tPA-SAMH-asd expresses SAMH gene fusing with AMH and hepatitis B surface antigen S gene. A large number of plasmids were extracted by the large-scale endotoxin removal kit *(Tiangen Biotech, China)* under the guide of instruction.

A total of 92 5-week-old female Kunming mice were purchased from the laboratory animal center of Huazhong Agricultural University, and raised in air-conditioned room at a constant temperature of 25°C with 12-h light/dark cycles, provided *ad libitum* with water and food. After 1 week of pre-feeding period, mice were randomly divided into four groups with 23 mice in each group. As shown in Table 2, mice from all the four groups were intramuscularly injected with 50 μg of pVAX-tPA-SAMH-asd (T1), 50 μg of pVAX-tPA-SINH-asd (T2), 25 μg of pVAX-tPA-SAMH-asd + 25 μg of pVAX-tPA-SINH-asd (T3), and 100 μl of physiological saline (C), respectively. A booster immunization was performed after 2 weeks of primary immunization. All mice were weighed and recorded before immunization, and 2 and 4 weeks following primary immunization. Blood was subsequently collected from the tail vein of each mouse and then centrifuged at 3,000 rpm for 10 min. The upper plasma was carefully separated and stored at −20°C for further use.

**Table 2:** Immunization program.

**Group**	**No**.	**Plasmid**	**Immunization dose**
T1	23	pVAX-tPA-SAMH-asd	50 μg/100 μl
T2	23	pVAX-tPA-SINH-asd	50 μg/100 μl
T3	23	pVAX-tPA-SAMH-asd and	2 × 25 μg/100 μl
		pVAX-tPA-SINH-asd	
C	23	Physiological saline	100 μl

### Measurement of Antibody Titers by ELISA

The specific anti-AMH and anti-INH antibodies in the plasma of immunized mice were detected by indirect ELISA. Briefly, the wells were coated with 50 ng/100 μl of either AMH or INH standard antigen *(Servicebio, China)* and incubated at 4°C overnight. After washing with PBS supplemented with 0.1% Tween-20 (PBST) for four times, the plates were blocked with 1% (w/v) BSA solution for 2 h at 37°C. Subsequently, 100 μl of serial diluted plasma (1:25, 1:50, 1:100, 1:200, 1:400, 1:800, and 1:1,600 dilution) was added into the wells and incubated at 37°C for 1 h. After three washes, antibodies were detected by adding HRP-labeled goat anti-mouse IgG antibody *(Servicebio, China)* for 1 h at 37°C. After that, 100 μl of TMB substrate *(Servicebio, China)* was added into each well for 15 min in dark; the reaction was then stopped by the addition of 2 M H_2_SO_4_ (50 μl/well), and OD values was determined at 450 nm by using a microplate reader (Bio-Rad, USA).

### Determination of Estrogen and Progesterone in the Plasma

Vaginal smears of mice were employed to determine estrous cycle at 2 weeks after booster immunization. Three mice in estrus period were selected from each treatment group to perform blood collection and ovary removal. The levels of steroid hormones in the plasma were detected by using estradiol *(Cusabio, CSB-E05109m, China)* and progesterone *(Cusabio, CSB-E05104m, China)* ELISA kits. The assay sensitivity was 40 pg/ml for estradiol and 0.2 ng/ml for progesterone.

### Evaluation of Litter Size and Offspring Birth Weight

After 4 weeks of primary immunization, all female mice were mated with healthy male mice. Mice were checked for the presence of a vaginal plug in the next morning. The male mice were picked out until the female mice were all pregnant. The litter size and offspring birth weight were carefully recorded.

### Calculation of Organ Index

At the end of the experiment, the mice were immediately sacrificed by cervical dislocation, and the major organs including heart, liver, spleen, lung, and kidney were collected and weighed. The organ indexes (organ weight/body weight) of these organs were calculated.

### Statistical Analysis

Each experiment was conducted at least in triplicate. Data analysis was performed using GraphPad Prism version 8.0 software and included one-way ANOVA followed by either Tukey or Bonferroni *post hoc* test.

## Results

### Effect of Inhibin A and Anti-Müllerian Hormone Alone on Estradiol and Progesterone Production in Granulosa Cells

**Figure 1:**
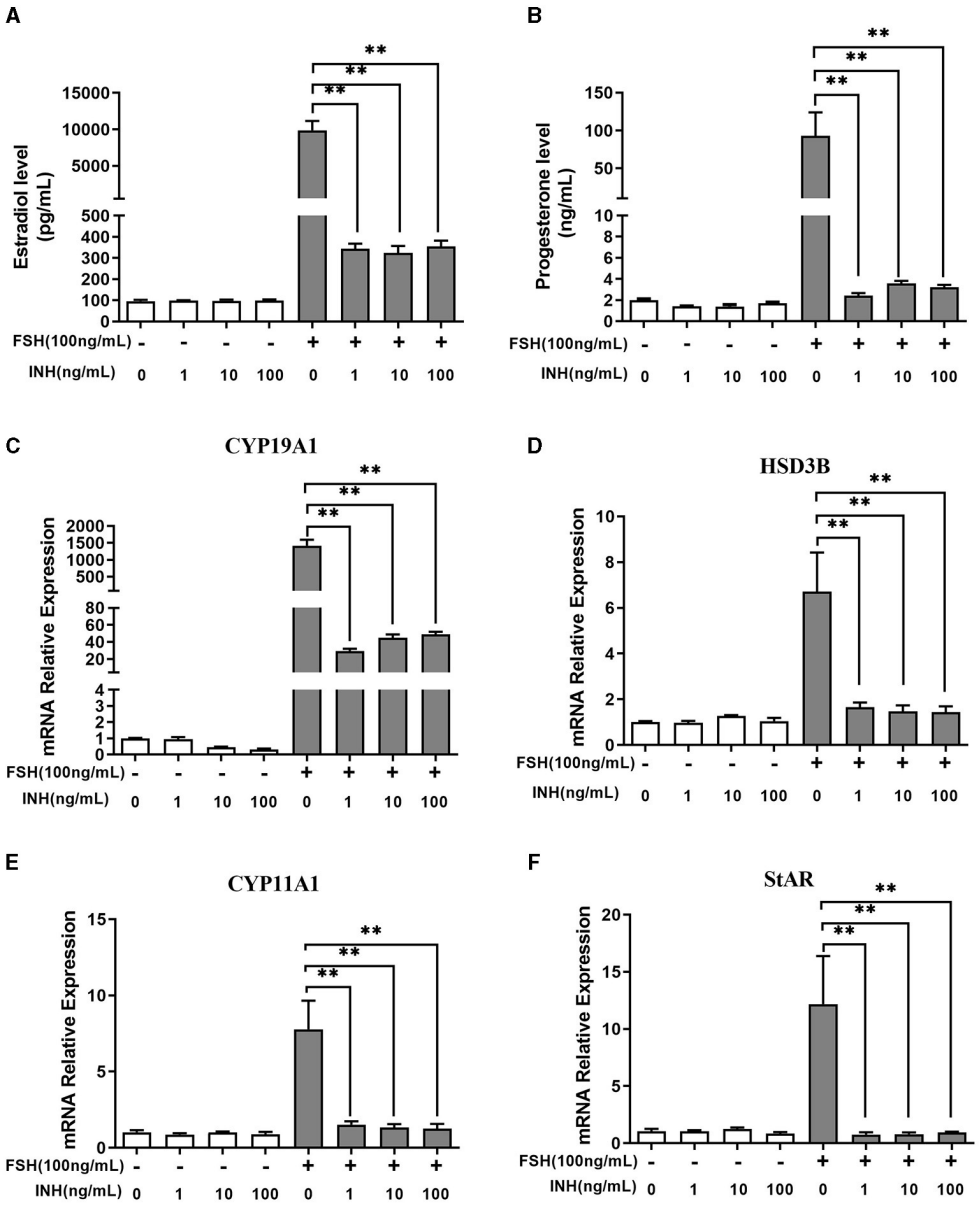
Effects of Inhibin A on the biosynthesis and secretion of estradiol and progesterone by primary granulosa cells. **(A,B)** Estradiol and progesterone production after treatment with increasing concentrations of INHA in the presence or absence of 100 ng/ml of follicle-stimulating hormone (FSH). **(C–F)** Relative expression of *CYP19A1, HSD3B, CYP11A1*, and *StAR* transcripts after treatment with increasing concentrations of INHA in the presence or absence of 100 ng/ml of FSH. Data are presented in mean ± SEM. **p < 0.01 between the indicated groups.

**Figure 2:**
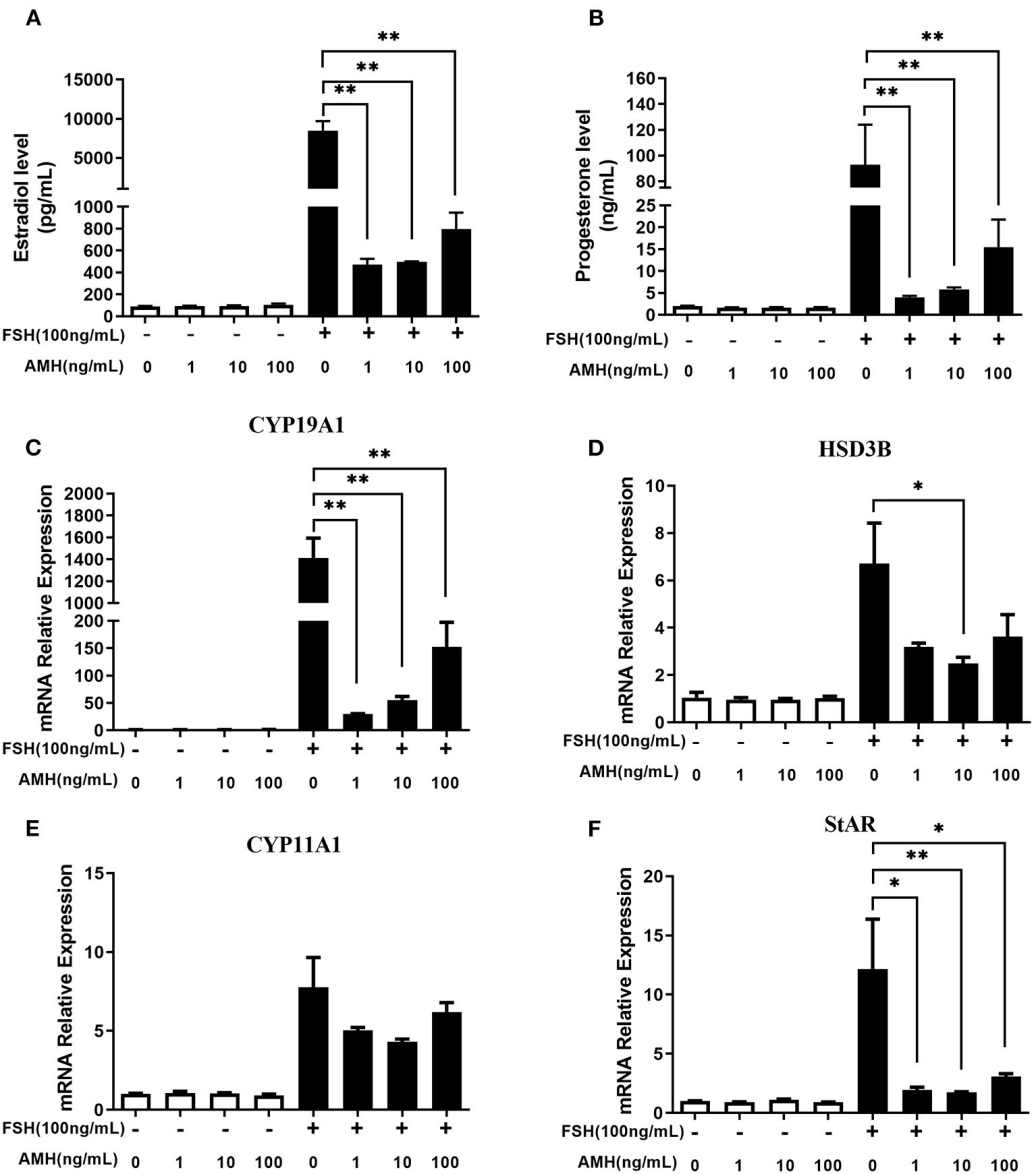
Effects of anti-Müllerian hormone (AMH) on the biosynthesis and secretion of estradiol and progesterone by primary granulosa cells. **(A,B)** Estradiol and progesterone production in cells treated with increasing concentrations of AMH in the presence or absence of 100 ng/ml FSH. **(C–F)** Relative expression of *CYP19A1, HSD3B, CYP11A1*, and *StAR* transcripts in cells treated with increasing concentrations of AMH in the presence or absence of 100 ng/ml of FSH. Data are presented in mean ± SEM. *p < 0.05 and **p < 0.01 between the indicated groups.

The results showed that INHA had no influence on the basal estradiol and progesterone production, whereas INHA in all concentrations significantly attenuated FSH-induced estradiol and progesterone production (*p* < 0.01; Figures 1A,B). Accordingly, INHA significantly decreased the mRNA expression of FSH-induced steroidogenic-related genes of *CYP19A1, HSD3B, CYP11A1*, and *StAR* (*p* < 0.01), but there was no significant effect of INHA on basal mRNA expression of *CYP19A1, HSD3B, CYP11A1*, and *StAR* transcripts (Figures 1C–F). Similarly, AMH significantly decreased the FSH-stimulated estradiol and progesterone production in a concentration-dependent manner (*p* < 0.01) but had no effect on basal estradiol and progesterone release (Figures 2A,B). In addition, AMH significantly reduced FSH-induced *CYP19A1, HSD3B*, and *StAR* mRNA expression, but not *CYP11A1* (Figures 2C–F).

### The Synergistic Effect of Inhibin A and Anti-Müllerian Hormone on Estradiol and Progesterone Production in Granulosa Cells

**Figure 3:**
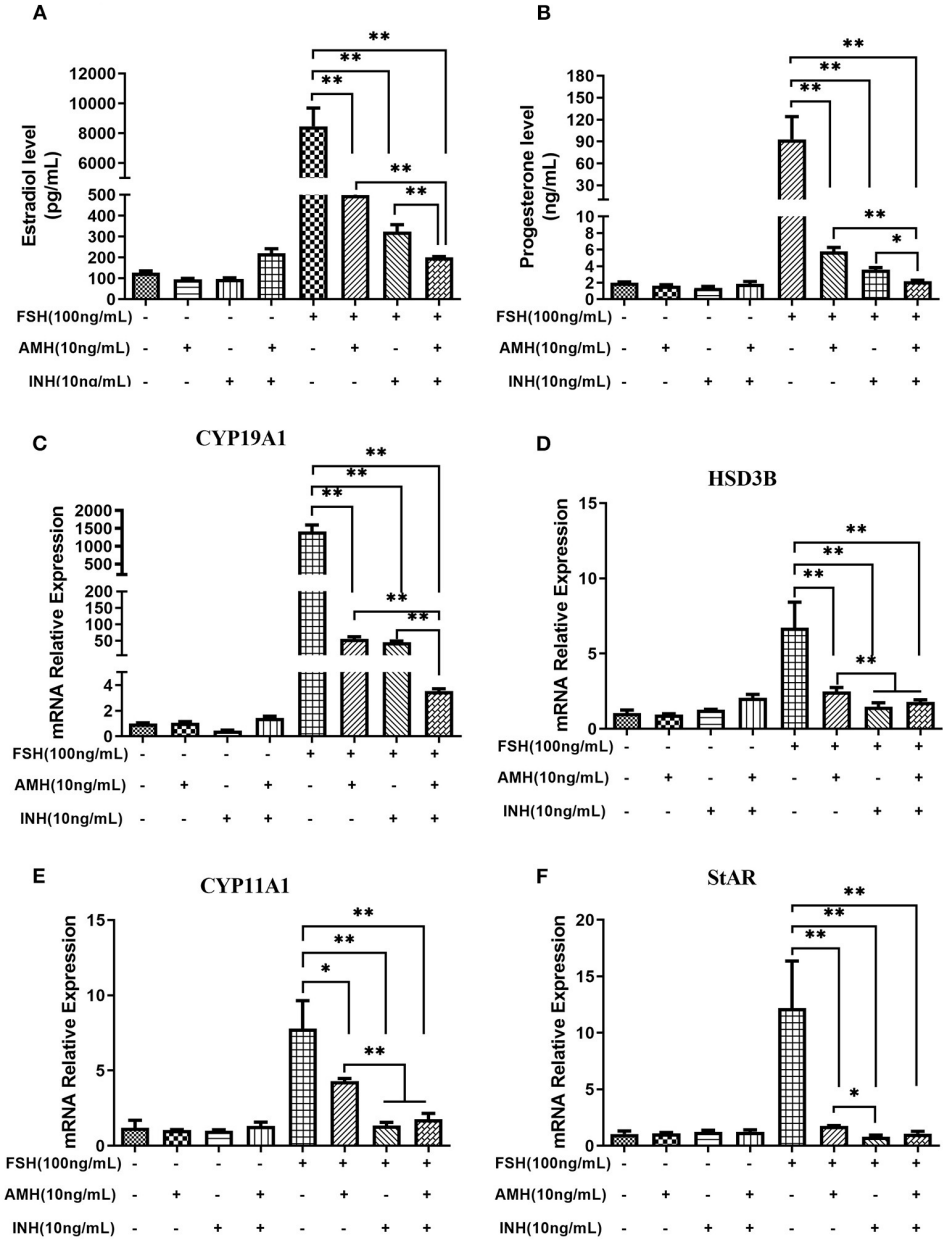
Synergistic effects of INHA and AMH on steroidogenesis by primary granulosa cells. **(A,B)** Estradiol and progesterone production in cells treated with INHA, AMH, or FSH alone or their combinations. **(C–F)** Relative expression of transcripts for *CYP19A1, HSD3B, CYP11A1*, and *StAR* in cells treated with INH, AMH, or FSH alone or their combinations. Data are presented in mean ± SEM. *p < 0.05 and **p < 0.01 between the indicated groups.

The results revealed that the treatment of AMH and INHA in combination or alone significantly inhibited FSH-stimulated estradiol and progesterone production (*p* < 0.01), but unaffected estradiol and progesterone production in the absence of FSH. Importantly, we found that AMH and INHA have a significant additive action in the inhibitory effect of FSH-induced estradiol and progesterone levels (Figures 3A,B). Furthermore, we also investigated the interaction between the expression of INHA and AMH showing that there was no significant difference on AMH levels in the supernatant after treatment with INHA, and *vice versa* (data not shown).

Accordingly, we observed that the treatment of AMH and INHA in combination or alone significantly downregulated FSH-stimulated mRNA expression of *CYP19A1, HSD3B, CYP11A1*, and *StAR* transcripts (*p* < 0.01; Figures 3C–F), but had no effect on basal expressions. In particular, real-time PCR results showed that the combined treatment of AMH and INHA significantly diminished FSH-induced mRNA expression of *CYP19A1* gene in granulosa cells than AMH or INHA alone (*p* < 0.01). Moreover, the FSH-induced mRNA expressions of *HSD3B, CYP11A1* genes in AMH and INHA combination group were significantly downregulated when compared with AMH alone group.

### The Synergistic Effect of Inhibin A and Anti-Müllerian Hormone on cAMP Levels in Granulosa Cells

**Figure 4:**
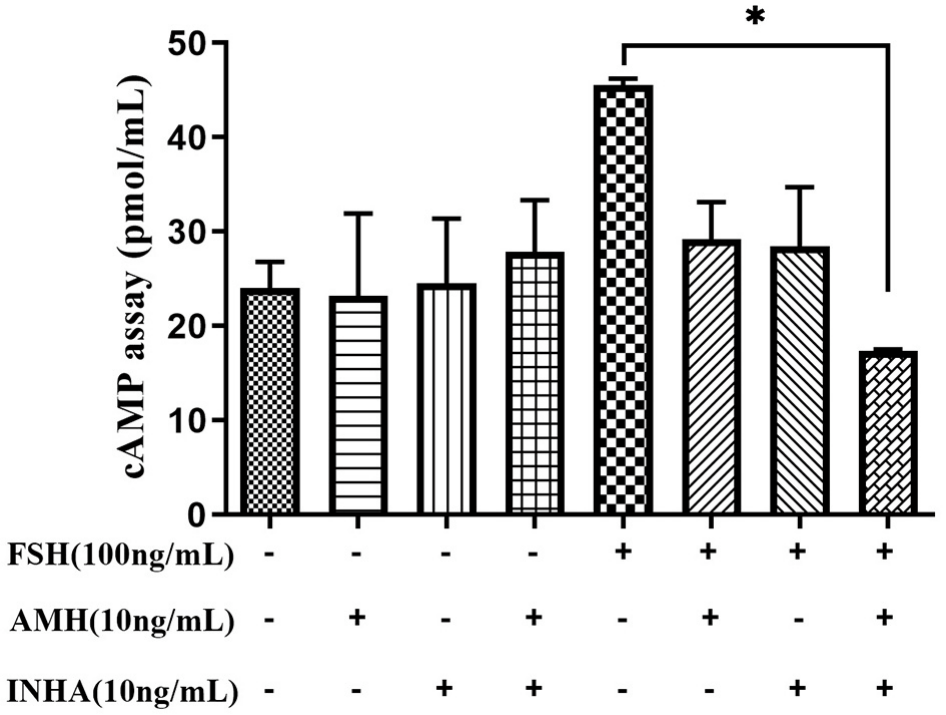
Effects of INHA, AMH, or FSH alone or their combinations on the content of intracellular cAMP in primary granulosa cells after 30 min of culture. Data are expressed as mean ± SEM, *p < 0.05 between the indicated experimental conditions.

The results of cAMP synthesis showed that there was no significant difference in each group at the basal level. However, FSH treatment significantly increased the intracellular cAMP level, while AMH and INHA combined treatment significantly inhibited the cAMP level induced by FSH (*p* < 0.05; Figure 4).

### Evaluation of Antibodies After Co-immunization of Inhibin and Anti-Müllerian Hormone Plasmids

After immunization with plasmids of pVAX-tPA-SINH-asd and pVAX-tPA-SAMH-asd, the levels of anti-AMH or anti-INH antibodies in plasma were detected at 4 weeks after the first immunization. As shown in Table 3, we found that all plasmids induced, respectively, specific antibodies. In particular, both anti-AMH and anti-INH antibodies were observed in T3 group, suggesting immunization program is successful in this experiment.

**Table 3:** Anti-Müllerian hormone (AMH) and inhibin (INH) antibody titers.

**Group**	**T1**	**T2**	**T3**
AMH antibody	1,260.4 ± 571.6	–	1,274.1 ± 520.8
INH antibody	–	1,403.6 ± 402.3	1,377.8 ± 434.4

*All data are represented in mean ± SD*.

### Effect of Co-immunization of Inhibin and Anti-Müllerian Hormone Plasmids on Estradiol and Progesterone Secretion

**Figure 5:**
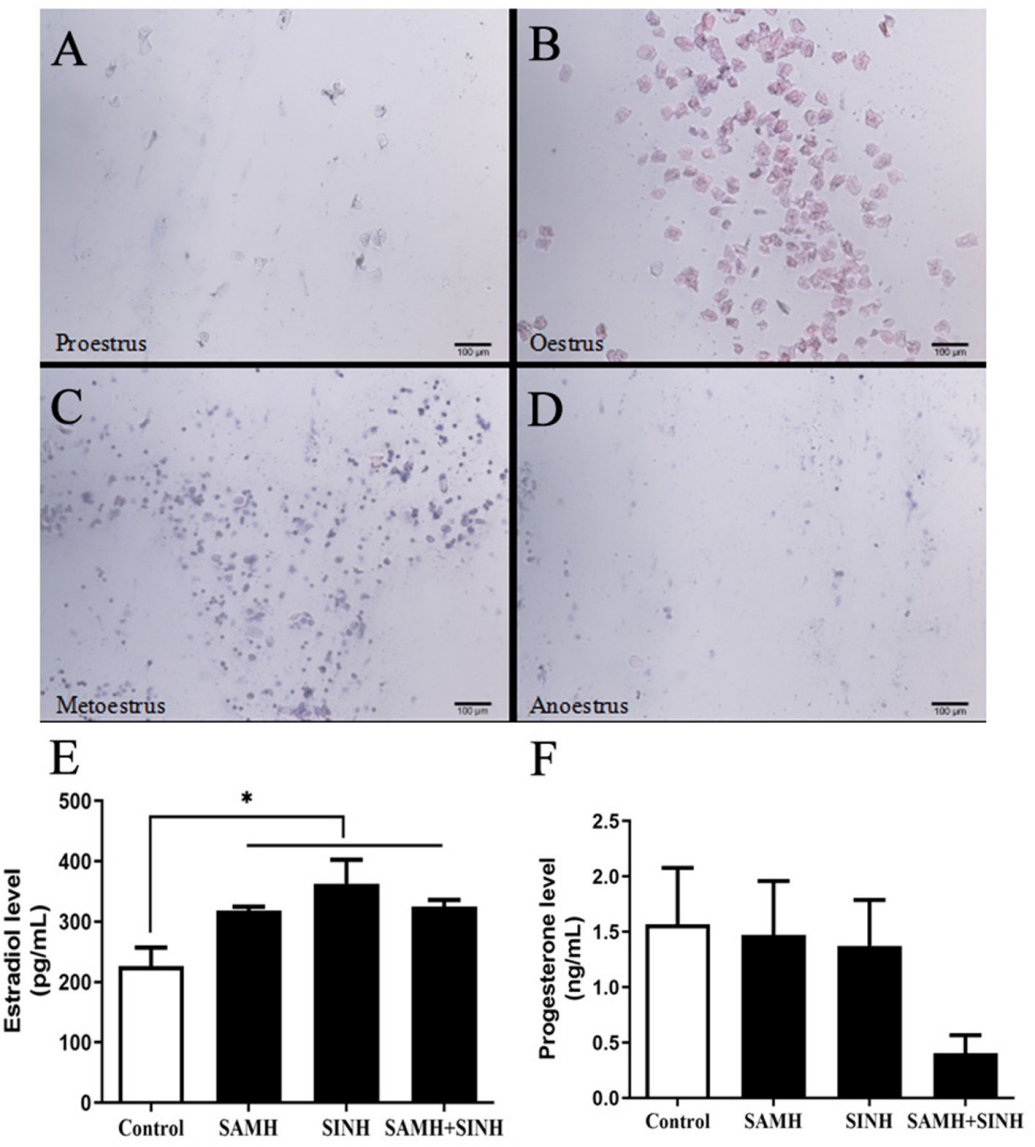
Effects of inhibin (INH) and AMH plasmids co-immunization on levels of estradiol and progesterone. **(A–D)** Vaginal smears of mice at the stages of the estrous cycle, the scale bar represents 100 μm. **(E,F)** Changes in steroid hormone of mice in estrus at week 4 after the primary immunization. Data are expressed as mean ± SEM, *p < 0.05 between the indicated experimental conditions.

We confirmed that the immunized mice indeed exhibited estrous as detected by vaginal smears (Figures 5A–D), and estradiol contents in all immunized group were significantly higher than that in the control group (Figure 5E). As for progesterone level (Figure 5F), there was no significant difference among all experimental groups, even though the decreasing trend was observed in the AMH plus INH immunized group.

### Effect of Co-immunization of Inhibin and Anti-Müllerian Hormone Plasmids on Litter Size in Mice

As shown in Table 4, the mean litter size in pVAX-tPA-SINH-asd and pVAX-tPA-SAMH-asd co-immunization group was significantly higher than that in control group. More specifically, the combined group obtained the highest litter size of 15.45, which increased by 1.6 compared with the control group. In addition, there was no significant difference in the offspring's birth weight.

**Table 4:** The changes of litter size and offspring birth weight in mice immunized with different plasmids.

**Group**	**Litter size**	**Birth weight (g)**
T1	14.10 ± 2.64^ab^	1.86 ± 0.19
T2	13.90 ± 2.15^a^	1.90 ± 0.12
T3	15.45 ± 1.76^b^	1.85 ± 0.16
C	13.85 ± 2.06^a^	1.89 ± 0.16

*All data are represented in mean ± SD. The values with different superscript letters in the same column are significantly different (p < 0.05)*.

### Effect of Co-immunization of Inhibin and Anti-Müllerian Hormone Plasmids on Organ Index

At the end of the experiment, the organ indexes (organ weight/body weight) of the heart, liver, spleen, lungs, and kidneys were calculated and compared. As shown in Table 5, there was no significant difference in the organ indexes between immunized group and control group, suggesting that the vaccines did not cause any adverse effect to mice.

**Table 5:** Organ index of mice immunized with different plasmids (100×).

**Group**	**Heart**	**Liver**	**Spleen**	**Lung**	**Kidney**
T1	0.50 ± 0.06	4.26 ± 0.53	0.34 ± 0.17	0.63 ± 0.06	0.99 ± 0.13
T2	0.56 ± 0.10	4.92 ± 0.58	0.37 ± 0.09	0.81 ± 0.16	1.07 ± 0.11
T3	0.59 ± 0.14	4.53 ± 0.97	0.34 ± 0.10	0.78 ± 0.10	0.96 ± 0.22
C	0.55 ± 0.11	4.41 ± 0.45	0.33 ± 0.06	0.76 ± 0.13	0.99 ± 0.08

*All data are expressed as mean ± SD*.

## Discussion

The growth and development of ovarian follicles are well-known to be modulated by gonadotropins, steroids, and local intraovarian signaling system. In response to FSH, granulosa cells secrete serial growth factors as coregulators of follicular development. Among them, both AMH and INH are members of the TGF-β superfamily and play crucial roles in folliculogenesis, acting in an autocrine or paracrine manner. It has been reported that AMH or INHA alone are able to inhibit the biosynthesis and secretion of estradiol induced by FSH in granulosa cells (6, 21–23), but the results are inconsistent (11), which are likely due to differences in species, culture medium, and hormonal concentrations. Moreover, there is compelling evidence of negative correlation between AMH and INH in human follicular fluid (31). Based on the above observations for similarity and specificity of AMH and INHA, we assume that INH may interact with AMH in regulating ovarian function. By combining both *in vitro* and *in vivo* studies, the present study reveals the synergistic effect of INH and AMH on follicular FSH responsiveness and fertility in mice.

In primary granulosa cell culture system, we found that both AMH and INHA alone significantly inhibited the secretion of FSH-induced estradiol and progesterone, and such inhibitory effect was exerted by inhibiting the mRNA expression of FSH-induced *CYP19A1, CYP11A1, HSD3B*, and *StAR*, which are important enzyme genes involved in steroid biosynthesis. These results are consistent with the previous studies from human granulosa cells (32, 33), which showed that AMH decreases sensitivity to FSH through reduction of the P450 aromatase activity, as well as, studies on cultured rat granulosa cells showing that INHA inhibits FSH-induced progesterone and estradiol production by decreasing P450 aromatase and P450 scc levels (6). Interestingly, it was found for the first time that INHA and AMH in combination had an additive inhibitory effect on FSH-induced estradiol and progesterone release, indicating that AMH and INHA play synergistic roles on steroidogenesis at the cellular level. We further demonstrated that the synergistic effect might be due to the downregulation of intracellular cAMP levels, which attenuates FSH-dependent cAMP/PKA steroidogenic pathway (34, 35). Within TGF-β superfamily, it has been demonstrated that AMH and BMP15 have the synergistic action on FSH-induced steroidogensis in human luteinizing granulosa cells possibly *via* smad5 protein phosphorylation (22). Apart from targeting gonads, AMH has been reported to synergistically cooperate with activin but not with BMP2 increasing FSHb expression in LβT2 cells (36, 37). In this respect, it would be interesting in the near future to analyze interaction of AMH and INHA in gonadotropin cells.

Immunological neutralization of reproductive hormone has been considered as a promising approach to regulate fertility. Our previous work has shown that INH DNA vaccine effectively reduced the biological activity of INH, thereby increasing the litter size (38, 39). Likewise, the synthesized AMH peptide can also activate the immune system of sheep, resulting in a significant decrease in the number of gonadotropin responsive preantral and small antral follicles, while significantly increasing the number and ovulation rate of gonadotropin dependent antral follicles (40). To our knowledge, this is the first report on co-immunization of AMH and INH DNA vaccine for improving the fertility in mice, even though there has been published a novel DNA vaccine harboring INH and the RFRP genes (39). We found that co-immunization with AMH and INH plasmids were able to induce both anti-AMH and anti-INH antibodies, which were proposed to neutralize endogenous AMH and INH, resulting in higher litter size. In Dan's study (39), the litter size of the first generation in mice increased by 14.71 after immunization with INH and RFRP dual expression plasmids p-TPA-SINH/TPA- SRFRP, which is less than the litter size in the current study. This can be explained by the fact that the mice used in our study have higher basal fertility rate, which could also be the possible reason for no significant change in fertility after INH immunization. In contrast to an earlier study on ewe (41), where significantly a higher number of lambs was born with low birth weight after INH immunization, we presented the increased litter size in the co-immunization group with no significant difference in offspring birth weights.

The plasma estradiol concentration in co-immunization of AMH and INHA group was significantly higher than that of control group, which is in agreement with a previous study in the rats indicating that INH DNA immunization induced higher estradiol concentrations than the control group (39). On the other hand, the changes in estradiol concentration *in vivo* are basically consistent with the synergistic regulation of AMH and INHA on steroid production at the cellular level. However, this finding does not apply to progesterone hormone since these mice were in estrus phase, that is to say, mature follicles did not ovulate, and CL formation did not occur (39). It is worth noting that AMH has been reported to induce FSHb expression and FSH secretion only in immature females, without any significant change in estradiol (37). It seems that AMH acts directly on FSH secretion specifically in females before puberty. Further studies in this direction are necessary in order to determine the effect of AMH on FSH expression and secretion in mature mice.

## Conclusion

We uncover that INHA and AMH synergistically inhibit FSH-induced estradiol and progesterone production by decreasing intracellular cAMP levels in primary granulosa cells. Furthermore, such synergistic action has been further confirmed through co-immunization with AMH and INH plasmids, showing higher estradiol levels and litter size in mice. Further studies on the underlying mechanism between AMH and INH signaling are essential for the elucidation of synergistic action in fertility of mice.

## Data Availability Statement

The original contributions presented in the study are included in the article/Supplementary Material, further inquiries can be directed to the corresponding author/s.

## Ethics Statement

The animal study was reviewed and approved by Scientific Ethics Committee of Huazhong Agricultural University (HZAUMO-2018-021).

## Author Contributions

All authors listed have made a substantial, direct and intellectual contribution to the work, and approved it for publication.

## Funding

This work was funded by the National Nature Science Foundation of China (31772604, 31772602, and 32072729).

## Conflict of Interest

The authors declare that the research was conducted in the absence of any commercial or financial relationships that could be construed as a potential conflict of interest.

## Publisher's Note

All claims expressed in this article are solely those of the authors and do not necessarily represent those of their affiliated organizations, or those of the publisher, the editors and the reviewers. Any product that may be evaluated in this article, or claim that may be made by its manufacturer, is not guaranteed or endorsed by the publisher.
