# Supplementary figures and images for: Synergistic Regulatory Effect of Inhibin and Anti-Müllerian Hormone on Fertility of Mice
Source: Front Vet Sci. 2021 Oct 28;8:747619. doi: 10.3389/fvets.2021.747619 (PMC8607300; doi:10.3389/fvets.2021.747619)

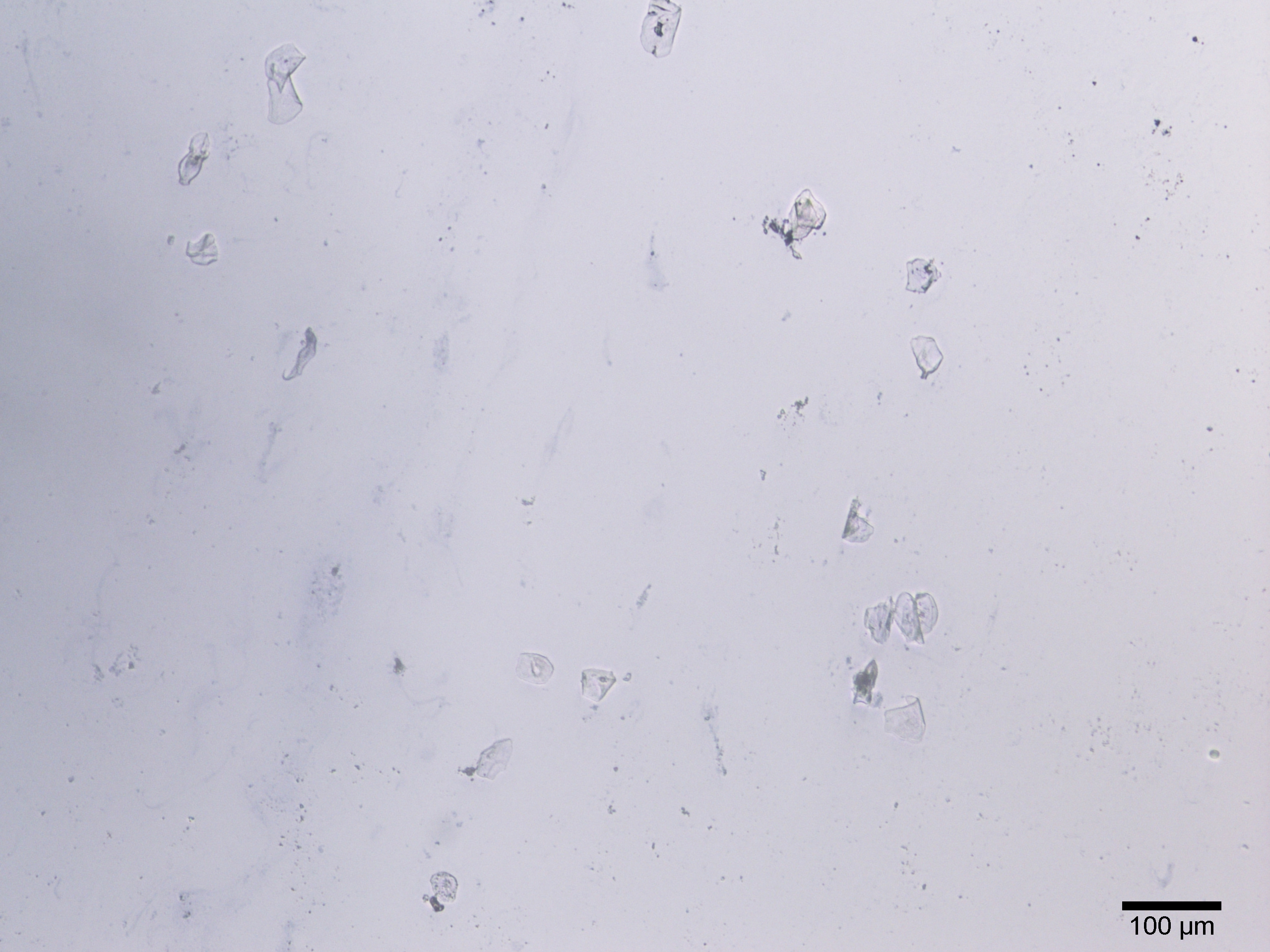

Supplement: Supplementary file 1 [file Image_1.JPEG]

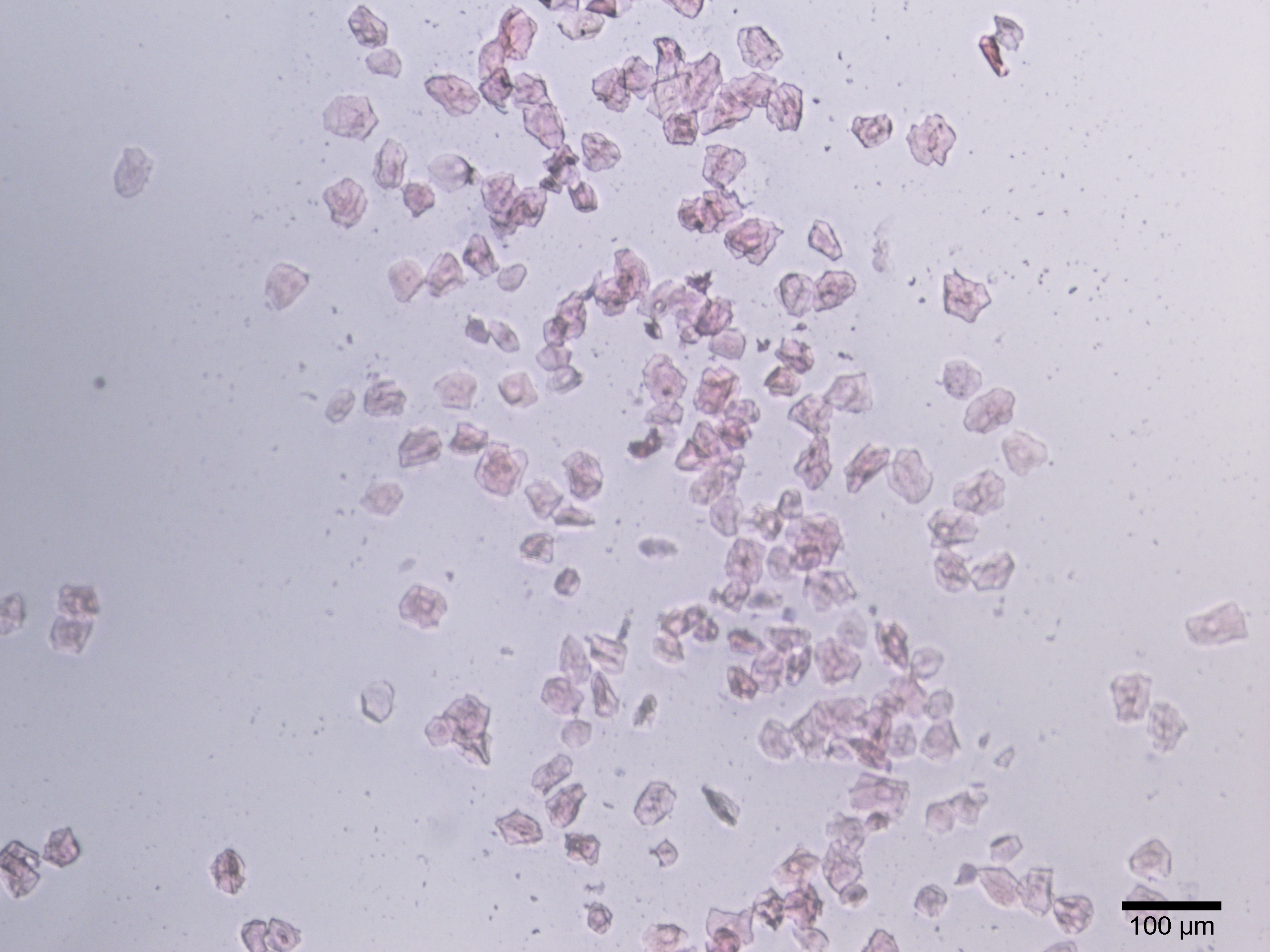

Supplement: Supplementary file 2 [file Image_2.TIF]

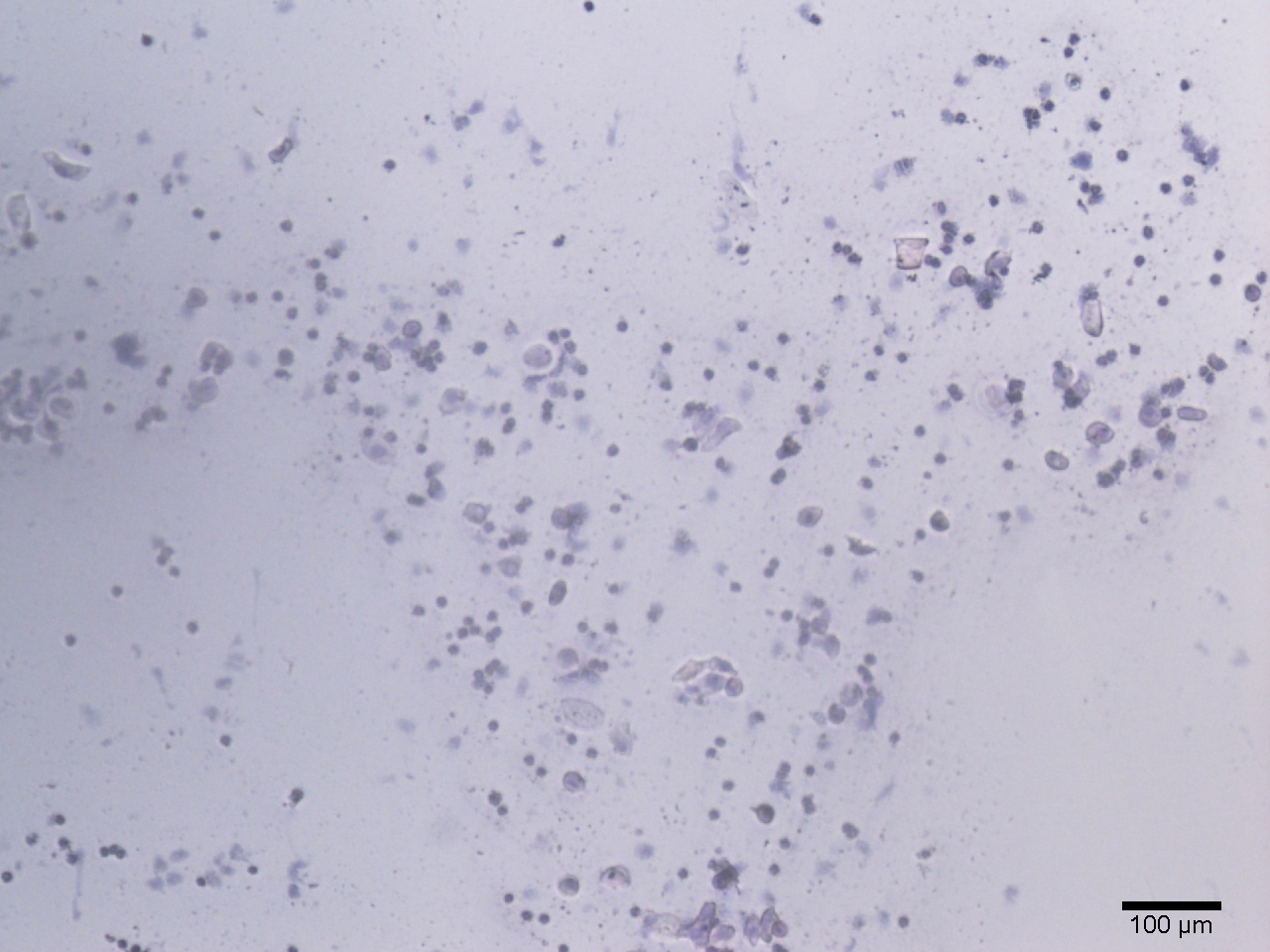

Supplement: Supplementary file 3 [file Image_3.JPEG]

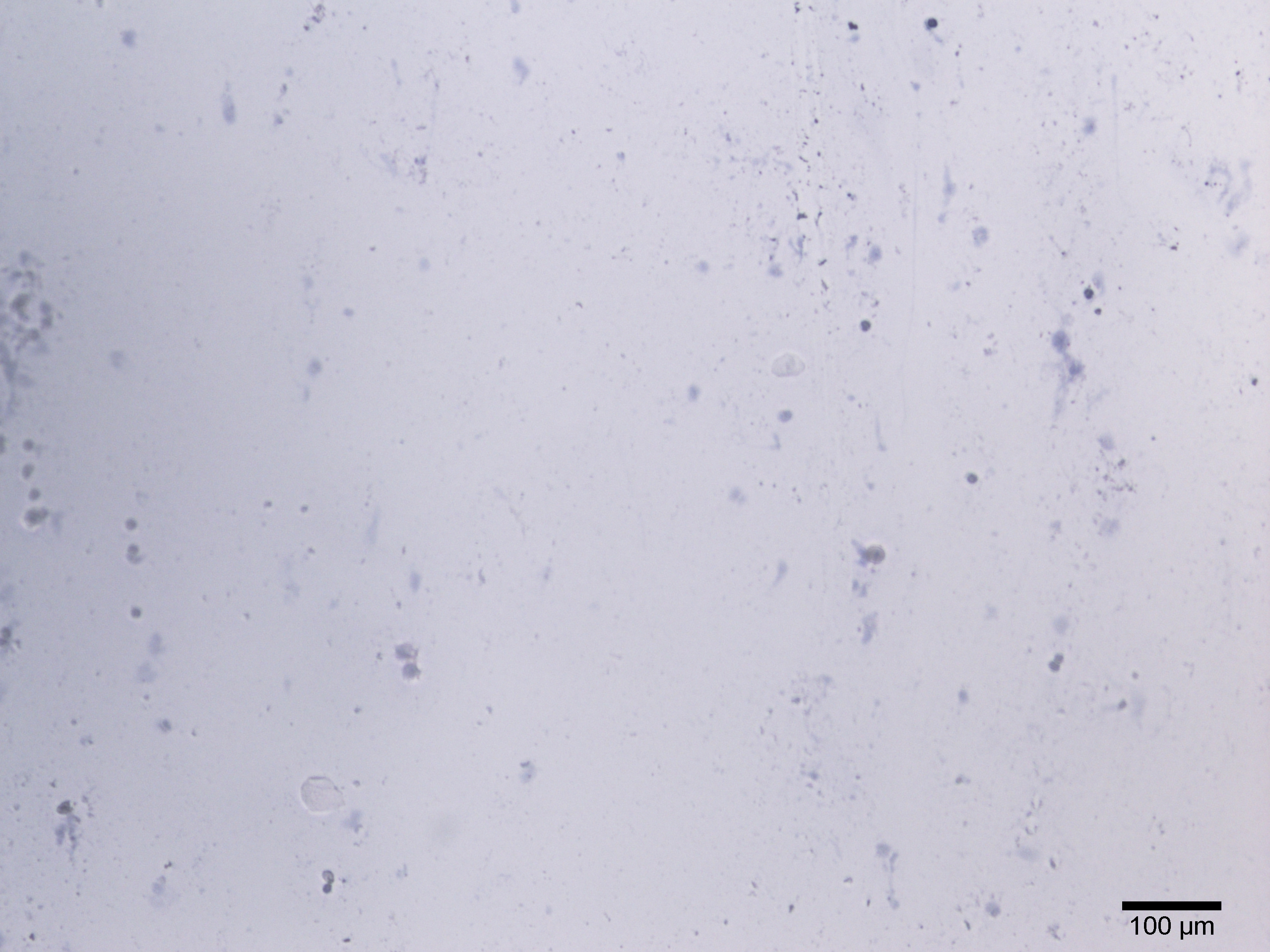

Supplement: Supplementary file 4 [file Image_4.JPEG]
